# Supplementary material for: Are carbon emissions trading and green financial instruments synergistic? -Comprehensive quantitative research based on content analysis
Source: PLoS One. 2024 Mar 7;19(3):e0298601. doi: 10.1371/journal.pone.0298601 (PMC10919601; doi:10.1371/journal.pone.0298601)
Supplement: S1 Data — (ZIP) [file pone.0298601.s001.zip › Data/地级市Z_F工作报告.zip!/║╙─╧/Σ≡║╙╩╨/└┤╫╘╣1⁄2╓┌║┼í╛┬φ┐╦╩2╛▌═°í┐_2012.docx]

各位代表:

现在，我代表市人民政府，向大会报告政府工作，请予审议，并请各位政协委员和其他列席人士提出意见。

折叠编辑本段工作回顾

五年来，面对复杂形势和严峻挑战，市政府在省委、省政府和市委的正确领导下，在市人大、市政协的监督支持下，深入贯彻落实科学发展观，团结带领全市人民，抢抓机遇，攻坚克难，开拓创新，奋力拼搏，完成了市五届人大一次会议确定的目标任务，经济社会发展取得了显著成就。

--经济实力跨上新台阶。五年来，我们始终坚持以经济建设为中心，全力以赴保增长、调结构、促转型，实现了经济又好又快发展。2011年，全市生产总值767.1亿元、财政总收入67.2亿元、一般预算收入33.7亿元、全社会固定资产投资448.1亿元、社会消费品零售总额254.1亿元，均比2006年翻一番;城镇居民人均可支配收入16998元、农民人均纯收入7700元，分别比2006年增加7964元和3817元。三次产业结构进一步优化，二三产业比重达到87.6%。食品、造纸、盐化工主导产业特色优势凸显，电子信息等新兴产业发展迅速，现代物流业日益壮大。全市6个省级产业集聚区入驻各类企业650家，2011年主营业务收入突破1000亿元、实现税收突破20亿元，经济开发区、临颍产业集聚区进入全省先进行列。

--改革开放迈出新步伐。五年来，我们始终坚持以改革增强动力、以开放激发活力，强力推进改革创新和开放招商，坚持不懈抓好重点项目建设，发展动力活力不断增强。各项改革不断深化，90家企业通过战略重组焕发生机和活力;成功组建发投、房投、西城投等投融资平台，在全省第一家发行城投债券，市商业银行挂牌运营，农村信用社达标升级，金融生态环境不断优化;财税、粮食、户籍等方面的改革深入推进;政府机构改革、事业单位改革、行政审批制度改革取得新成效。对外开放水平全面提升，累计实际利用外资14.1亿美元、利用省外资金356.4亿元，累计新引进企业754家，其中世界500强8家、国内500强14家、行业百强28家。重点项目建设成果丰硕，累计建成252个，完成投资486亿元。五年来，连续成功举办中国(漯河)食品博览会，会展的国际化、市场化水平不断提升，我市食品名城的品牌效应更加彰显，漯河在国际、国内的知名度和影响力进一步扩大。

--"三农"工作取得新成效。五年来，我们始终坚持把"三农"工作作为重中之重，各级财政累计投入"三农"资金52.9亿元，有力推动了农业增效、农民增收和农村发展。粮食总产连续八年创历史新高，标准化规模养殖场发展到573个，农业产业化龙头企业达到461家，农民专业合作社发展到752家。全市土地流转面积达到68万亩。集体林权制度改革走在全省前列。我市成功创建全国首家农业综合标准化示范市。农村生产生活条件明显改善，全市有效灌溉面积占耕地面积的比例达到91.6%，小麦生产全程实现了机械化，新建、改造农村公路1285公里，解决了65万农村人口饮水安全问题，农村户用沼气池发展到21.5万座，农村超市达到1249个。累计实现8.6万农村贫困人口脱贫解困。新型农村社区建设稳步推进，2100户农民喜迁新居。

--城乡面貌发生新变化。五年来，我们始终坚持城乡统筹发展，不断加快城乡基础设施建设，全面提高城乡管理水平，城市的吸引力和带动力不断增强。2011年，城镇化率达到40.9%，比2006年提高了9.3个百分点。大力度推进沙澧河开发建设，建成了435公顷的沿河景观带。漯河新区获得批准，西城区建设全面启动，市区建成区面积扩大到65平方公里，常住人口达到62万人。新建、改造市区道路70条，改造背街小巷142条，铺设供水管网76公里、燃气管网456公里、热力管网41公里，污水日处理能力达到21万吨，垃圾无害化处理率达到90%以上，城市绿化覆盖率达到38.6%，人均公共绿地面积12.6平方米。城市管理综合提升行动成效明显。全面实施"乡村清洁工程"，村容村貌得到较大改善。我市相继荣获中国优秀旅游城市、国家森林城市、省级文明城市、全省城市精细化管理优秀城市等称号。

--民生质量得到新提升。五年来，我们始终坚持以民为本，更加重视保障和改善民生，着力解决了一批群众最关心、最直接、最现实的利益问题，全市人民享受到了更多的改革发展成果。每年坚持实施民生工程，各级财政累计投入223亿元用于民生改善。城镇职工、城镇居民医疗保险参保人数分别达到32.4万人和40.6万人;新农合参合率达到98.1%，共有692万人次获得医疗补偿10.3亿元;城镇职工养老、失业、工伤、生育保险覆盖面持续扩大;新型农村养老保险成效明显，27.5万名六十岁以上的农民按月领取基本养老保险金;城乡低保和农村五保供养标准提高，与2006年相比，城市低保标准由每月的130元提高到260元，农村低保标准由每年的720元提高到1300元，农村五保集中供养标准由每年的1200元提高到2240元。建设各类保障性住房3.2万套。累计发放住房公积金贷款13.3亿元。累计新增城镇就业人员19万人、安置下岗失业人员再就业7.8万人、新增农村劳动力转移就业15万人，我市荣获"中国创业之城"称号。

--社会事业实现新发展。五年来，我们始终坚持经济社会协调发展，促进了各项社会事业繁荣进步。科技、人才工作得到加强，新建2个院士工作站和6个省级工程技术中心。义务教育均衡发展成效突出，"两免一补"政策全面落实，普通高考本科上线人数连年保持万人以上，漯河医专新校区一期建成投用。职业教育全省领先，职业技术学院荣获河南省示范性高职建设院校，民办食品职业学院申办成功，技师学院获得批准。改扩建了一批市、县医院和乡镇卫生院，建成了1258个标准化村卫生室、10个社区卫生服务中心、31个社区卫生服务站。我市连续五次荣获全国无偿献血先进市称号。文化旅游业发展加快，新创建3个国家3A级景区，新建47个乡镇综合文化站、1060个农家书屋，许慎文化园、陈星聚纪念馆建成开放。群众体育工作继续保持全国先进。移民安置和对口援建工作圆满完成。计划生育、环境保护和土地管理三大基本国策得到较好落实。节能减排目标全面完成，公共机构节能工作走在全省前列。审计、统计、人防、气象等工作进一步加强，广播影视、新闻出版、史志档案、民族宗教、外事侨务、慈善事业等取得新成效，妇女、未成年人、老年人和残疾人合法权益得到保障。驻漯解放军、武警官兵和民兵预备役在支持地方经济建设、维护社会稳定等方面作出了重要贡献，军民融合式发展进一步深化，我市荣获全国双拥模范城称号。

民主法制建设不断加强，自觉接受人大和政协监督，认真办理人大代表议案、建议和政协委员提案，依法治市和法治政府建设深入推进，廉政建设和反腐败斗争取得新成效。精神文明建设成效显著，我市成为全国中华文化经典诵读示范城市、全国未成年人思想道德建设先进市。社会大局和谐稳定，我市被评为全国社会治安综合治理优秀市、全国法制宣传教育先进市、全省安全生产优秀市、平安建设先进市和信访工作先进市。

各位代表!过去的五年，是我市综合实力显著增强的五年，是城乡面貌发生重大变化的五年，是人民生活水平明显提升的五年，实现了"十一五"精彩结局、"十二五"良好开局，漯河已站在新的历史起点上，进入了一个稳健发展的黄金期。这些成绩的取得，是省委、省政府和市委正确领导的结果，是市人大、市政协监督支持的结果，是全市人民团结拼搏的结果。在此，我代表市人民政府，向工作在全市各条战线上的工人、农民、知识分子、干部，向驻漯部队、武警官兵、公安民警以及各界人士，致以崇高的敬意!向所有关心支持漯河发展的同志们、朋友们，表示衷心的感谢!

回顾五年来的工作，我们深深体会到:只要始终坚持科学发展，集中精力抓工业，齐心协力抓招商，不遗余力抓项目，就一定能够不断增强经济实力和综合竞争力;只要始终坚持解放思想，敢于争当先锋，勇于改革创新，善于营造环境，就一定能够形成推动发展的强大合力;只要始终坚持以人为本，深入体察民情，充分尊重民意，切实改善民生，就一定能够赢得人民群众的信赖和支持;只要始终坚持务实重干、求实求效，不怕困难、敢于担当，科学运作、雷厉风行，就一定能够不断开创各项建设事业新局面，谱写漯河跨越发展新篇章!

各位代表!在肯定成绩的同时，我们也清醒地认识到，我市经济社会发展中还存在不少问题和不足:经济综合竞争力不强;产业结构不够合理，高新技术产业规模小，服务业发展薄弱;重大项目较少，支撑全市快速发展的后劲不足;一些地方经济发展环境尚不宽松。城镇化水平较低;城乡规划滞后;城市配套功能不完善，供热等基础设施不健全;城市公交、小区物业等方面的改革、整顿亟待加强。就业、医疗、上学、住房等民生改善方面与广大群众的期待尚有一定差距;弱势群体救助、失地农民生活保障等方面的机制还不健全。政府自身建设还存在薄弱环节，一些部门职能转变不到位、行政服务效能不高;精神懈怠、作风浮躁、贪图安逸、奢侈浪费等现象在个别干部身上明显存在。对此，我们一定高度重视，认真对待，采取有力措施，切实加以解决，决不辜负人民的期望。

折叠编辑本段目标任务

根据市六次党代会的战略部署，今后五年政府工作的总体要求是:坚持以科学发展为主题，以加快转变经济发展方式为主线，以质量立市为指导方针，以富民强市为根本目的，抢抓中原经济区建设重大机遇，围绕"一区两城一中心"战略目标，着力推进"三化"协调发展，着力调整优化经济结构，着力深化改革开放，着力保障和改善民生，努力保持经济又好又快发展和社会大局和谐稳定。

今后五年，我市经济社会发展的主要奋斗目标是:

--经济发展速度全省领先。到2016年，经济总量、地方公共财政预算收入、固定资产投资均比2011年翻一番。主要经济指标增速力争进入中原经济区前列。

--改革开放水平全省领先。重点领域和关键环节的改革实现重大突破，科学发展的体制机制新优势加快形成。开放型经济水平明显提升，形成全方位、多层次、宽领域的开放新格局，努力打造中原经济区开放新高地。

--"三化"协调发展全省领先。探索不以牺牲农业和粮食、生态和环境为代价的"三化"协调科学发展的路子，到2016年，城镇化率达到50%以上，构建以中心城区为龙头、临颍和舞阳两个县城为组团、10个中心镇为节点、50个新型农村社区为基点的现代城镇体系;形成以新型城镇化为引领、"三化"协调推进格局，创建中原经济区"三化"协调发展先行区。

--群众幸福指数全省领先。城乡居民收入高于全省平均水平，覆盖城乡居民的社会保障体系更加完善，基本公共服务均等化程度显著提高，社会更加和谐稳定，致力把漯河建成中原经济区幸福家园。

今后五年，重点抓好四大任务。

强力推动三次产业大发展。不断调整优化产业结构，做优一产、做强二产、做大三产，全面推动三次产业优化升级，提升壮大经济实力和综合竞争力。大力发展现代农业，着力实施高标准粮田"百千万"建设工程和现代农业产业化集群培育工程，加快解决农技推广、农信服务、农田水电路配套等"最后一公里"问题，实现农业农村服务网络全覆盖。到2016年，建成粮食万亩示范方40个、现代农业示范园区160个，粮食生产全程实现机械化，努力把我市打造成全省最具影响力的现代农业示范市。坚持工业强市战略不动摇，培育壮大骨干企业和主导产业，经过五年的努力，形成以千亿双汇为龙头，以一大批超50亿元、超10亿元企业为支撑的发展格局，努力打造两千亿食品产业、超千亿化工产业、超千亿以高新技术为主的战略性新兴产业，把漯河建成全国有影响力的食品生产基地、中西部地区最大的造纸基地、全省重要的盐化工基地和电子信息产业基地。加快发展服务业，提高第三产业占生产总值的比重，重点发展现代物流、文化旅游、餐饮娱乐、金融服务和房地产业，加快建设中心商务功能区和县区特色商业区，构建区域性商贸物流中心，形成大市场、大流通、大贸易格局。

强力推动招商引资和项目建设大突破。以更宽的视野拓展开放领域，以更优的服务营造开放环境，大力度抓好招商引资，高起点承接产业转移，吸引更多的知名企业入驻漯河、更多的外来资金投向漯河。到2016年，全市实际利用境外资金突破10亿美元、实际利用省外资金达到400亿元，年均增速均在20%以上。集中精力抓好重点项目建设，谋划实施一批投资额度大、科技含量高、带动能力强、市场前景好、税收贡献多的重大项目，带动全社会固定资产投资年均增速达到22%以上。以项目的大建设，拉动投资大增长，推动漯河大发展。

强力推动城市品位大提升。坚持以高端规划引领城市发展，以精致设计凸显城市个性，推动城市功能现代化、城市服务专业化、城市管理精细化，努力实现三年面貌大改变、五年品位大提升。继续高标准推进沙澧河开发建设，全面完成二期开发建设任务。加快推进漯河新区、西城区开发建设，进一步拉大城市框架。加快旧城区、城中村和背街小巷改造，五年内建成区城中村和背街小巷基本改造完毕。加快城市基础设施建设，努力实现建成区供水、供气、供热管网全覆盖。围绕打造区域性综合交通枢纽和现代商贸物流中心，构建以铁路、高速公路、干线公路、水运通道为骨架的大交通体系，实现漯河连南贯北、通江达海的夙愿。加快"数字漯河"管理平台建设，创新城市管理模式。积极创建国家卫生城市。到2016年，"双百"城市框架基本形成，滨水城市特色充分彰显，努力把漯河建成功能完善、环境秀美、品位高雅、生态宜居的中原时尚之都。

强力推动民生质量大提高。持续加大民生投入，继续实施民生工程，确保民生得到较大改善。着力破解"四个难题":五年累计新增城镇就业人员20万人，新增农村劳动力转移就业17万人，有效解决就业难问题;建设各类保障性住房5万套，有效解决城镇居民住房难问题;在全市新建各类幼儿园150所、小学17所、初中10所，有效解决入园难、入学难问题;深化医药卫生体制改革，完善城乡医疗卫生服务体系，有效解决群众看病贵、就医难问题。着力实现"四个全覆盖":进一步完善社会保障体系，实现城乡居民社会养老全覆盖;大力实施文化惠民工程，实现城乡公共文化设施全覆盖;拓宽计生服务领域，实现计划生育优质服务全覆盖;扎实开展城市老旧住宅小区环境综合整治，实现城市住宅小区物业管理全覆盖。着力提升"四个水平":严格执行最低工资标准，建立工资正常增长机制，深化全民创业行动，提升城乡居民收入水平;加大财政投入，实施社会救助，提升困难群众和弱势群体的生活保障水平;加快城市基础设施向农村延伸、城市公共服务向农村覆盖、城市现代文明向农村辐射，深化乡村清洁工程，提升城乡一体化水平;创新社会管理体制机制，完善基层社会管理和服务体系，提升社会管理科学化水平。

各位代表!目标催人奋进，漯河前程似锦。只要我们以更加强烈的紧迫感、使命感和责任感，务实重干，砥砺奋进，加速前行，在新一轮竞争中勇立潮头，在加快"三化"进程中争当先锋，在中原经济区建设中跨越发展，未来五年的宏伟蓝图就一定会变成美好现实，漯河的明天就一定会更加灿烂辉煌!

折叠编辑本段工作安排

今年是"十二五"时期承前启后的重要一年，也是新一届政府的开局之年。我们面临的外部环境更为复杂严峻。世界经济复苏的长期性、艰巨性和复杂性更加凸显，国际市场低迷、外需不足可能成为常态。我国经济增速回落与物价仍处高位相互交织，投资需求逐步放缓，居民消费增长难度加大，需求不足上升为经济运行主要矛盾。国内区域之间围绕市场、资金、资源、技术、人才等展开的竞争日趋激烈，我省各地抢抓中原经济区建设机遇、竞相发展的势头更为强劲，我市面临着更大挑战。同时也要看到，我市发展仍处于重要战略机遇期。国际、国内产业加快转移的趋势没有改变。国家继续实施积极的财政政策和稳健的货币政策，有利于我们更好地争取国家支持。随着国家支持中原经济区建设意见的出台，我市与省政府有关部门签订了一系列战略合作协议，我们将争取更多的项目和资金。经过近年来的发展，我市已处于工业化、城镇化快速发展阶段，发展基础更加坚实。尤其是温家宝总理视察漯河，极大鼓舞了全市上下的士气，更加坚定了我们加快发展的信心。我们一定要认清形势，抢抓机遇，坚持稳中求进、稳中求好、稳中求快，以更大的气魄、更强的力度、更有效的举措，持续保持好趋势、好态势、好气势，推动经济社会发展再上新台阶。

今年经济社会发展的主要预期目标是:生产总值增长12%，地方公共财政预算收入增长12%，全社会固定资产投资增长22%，实际利用外资增长20%,社会消费品零售总额增长18%，城镇居民人均可支配收入增长10%,农民人均纯收入增长10%，城镇新增就业4万人，人口自然增长率、居民消费价格涨幅控制在省定目标以内，省定节能减排目标全面完成。

围绕上述目标，今年要坚定不移地抓好六项重点工作。

(一)坚持以新型城镇化为引领，致力构建现代城镇体系。城镇化水平低是我市经济社会发展诸多矛盾的聚焦点，必须把加快推进新型城镇化作为改善民生、扩大内需、转变发展方式的综合性举措，统筹推进中心城市、县城、中心镇和新型农村社区建设。

。强化规划的龙头作用。坚持以全域理念完善规划体系，以精品意识提升规划水平，以铁的手腕严格执行规划。开展"规划提升年"活动，完成城市总体规划的评审和报批工作，编制完成中心城区控制性详规和各类专项规划，高水平规划设计城市重点区域和重要节点;深入推进村镇规划编制工作，完成10个重点镇主要街区控制性详规、50个新型农村社区规划编制。强化中心城区辐射带动作用。突出抓好沙澧河开发带、漯河新区、经济开发区、西城区"一带三区"建设，着力抓好投资60亿元的十大城建重点工程。深入推进沙澧河开发建设，圆满完成一期建设任务，科学规划设计二期工程，全面掀起建设热潮。加快漯河新区和西城区开发建设，漯河新区核心区建设全面展开，西城区高铁客运站、环城水系、城市规划馆等项目建设加快推进。按照一级公路标准，对107国道漯河境全线实施升级改造，将市区段改造成城市道路。新建、改造交通路、建设路、新东环等12条道路，规划建设中心城区外环快速通道，加宽、改造交通路沙河大桥，加快推进沙颍河复航工程及漯河港项目建设，新建、改造一批供排水、供气、供热工程。开工建设泰山路、舟山路等5座提排泵站，切实解决市区主干道积水问题。开工建设市污泥处理厂和3个产业集聚区污水处理厂，新建、改造星级公厕20座、垃圾中转楼5座。改造10个城中村、24条背街小巷。持续开展城市管理综合提升行动，全面整治交通秩序、经营秩序、市容市貌和违法建设，深化城市公交改革;加强住宅小区物业管理，综合整治10个老旧住宅小区。扎实推进城市精细化和网格化管理，建成城市数字化管理平台。建立郾城、源汇、召陵三区和经济开发区、西城区联席会议制度，定期研究解决发展中的重大问题，促进各区更好地协调发展。强化县城和重点镇承载承接作用。进一步明确临颍、舞阳两个县城的产业定位和功能分区，发挥特色优势，错位竞争发展。加快颍北新区、舞北新区建设，全面推进基础设施和公共服务设施建设，吸引产业和人口向县城集聚，促进产城互动发展。推进重点乡镇内涵式发展，加大基础设施建设力度，扩大城镇规模，完善城镇功能，强化产业支撑，增强辐射带动能力，加速人口向城镇集中，努力打造一批特色鲜明、产业突出、优势明显、功能完善的小城镇。强化新型农村社区战略基点作用。按照"分类指导、科学规划、群众自愿、就业为本、量力而行、尽力而为"的原则，坚持因地制宜，重点选择产业集聚区规划区内、城乡接合部、中心镇周边村庄率先推进。通过加大财政、信贷投入、实行以奖代补、整合涉农资金、引导社会资金投入，积极探索多元化投入机制，加快新型农村社区建设。从今年开始，每个县区每年建设2个入住5000人以上的新型农村社区，努力培育一批规划标准高、建设规模大、产业支撑强、配套功能全的新型农村社区。

(二)坚持以扩大开放为动力，致力抓好招商引资和重点项目建设。持续把招商引资作为"一号工程"，把扩大投资作为第一动力，锲而不舍，强力推进，努力实现招商引资和项目建设新突破。今年，新签约项目总额达到400亿元以上，当年完成投资200亿元以上。其中超亿元项目60个以上，超5亿元项目20个以上，超10亿元项目10个以上;重点项目建设投资200亿元以上，带动全市固定资产投资600亿元以上。着力提升招商水平。突出产业招商，围绕食品、化工、电子信息、装备制造等产业发展，大力引进龙头型、基地型企业，吸引配套企业和关联企业集群发展，加快做大做强特色产业集群。突出招大引强，紧盯世界500强、国内500强、央企、省企和行业龙头企业，加强重点联系，注重招商质量，力争再引进一批知名企业和重大项目。要高标准办好第十届中国(漯河)食品博览会，力争投资项目签约额、贸易合同签约额均超过200亿元。
